# Relationship between sleep quality and cardiovascular disease risk in Chinese post-menopausal women

**DOI:** 10.1186/s12905-017-0436-5

**Published:** 2017-09-11

**Authors:** Sek Ying Chair, Qun Wang, Ho Yu Cheng, Sally Wai-Sze Lo, Xiao Mei Li, Eliza Mi-Ling Wong, Janet Wing-Hung Sit

**Affiliations:** 1 The Nethersole School of Nursing, The Chinese University of Hong Kong, Esther Lee Building, The Chinese University of Hong Kong, Shatin, New Territories Hong Kong; 2 The Faculty of Nursing, College of Medicine, Xian Jiaotong University, Xi’an, Shaanxi Province China

**Keywords:** Cardiovascular disease risk, Post-menopausal women, Sleep quality

## Abstract

**Background:**

Menopause is an inevitable stage affecting every middle-aged woman. China has a large and increasing group of post-menopausal women. Most post-menopausal women suffer from increased risks for cardiovascular diseases (CVD) and sleep problems. Previous studies have demonstrated the associations between sleep disorders and increased CVD risks in general population. The current study is to examine the relationship between sleep quality and CVD risks among Chinese post-menopausal women.

**Methods:**

This study was a sub-study nested in a cross-sectional study that investigated the sleep quality of community-dwelling adults in Xian, Shaanxi Province, China. The Chinese version of the Pittsburgh Sleep Quality Index (PSQI) and the Framingham 10-year risk score (FRS) were used to measure sleep quality and CVD risk among 154 Chinese post-menopausal women. Multivariate regression and logistic regression were used to determine the association between sleep quality and CVD risk.

**Results:**

The participants (age: 63.65 ± 4.47 years) experienced poor sleep quality (mean score of global PSQI = 8.58) and a 10-year risk of CVD of 12.54%. The CVD risk was significantly associated with sleep duration (β = − 0.18, *p* = 0.04) and sleep disturbance (β = 0.33, *p* < 0.001). Women with good sleep quality (PSQI ≤5) were less likely to be at high risk for CVD (FRS > 10%) (odds ratio = 0.51, *p* = 0.04).

**Conclusions:**

Poor sleep quality might increase the CVD risk in post-menopausal women. Interventions to promote the cardiovascular health of Chinese post-menopausal women may need to include sleep promotion strategies.

## Background

Menopause is a natural and an inevitable stage leading to old age that affects every middle-aged woman. Every day, about 6000 American women reach menopause [1]. In China, 120 million women, accounting for around 23% of the world’s menopausal population, are experiencing the transition to menopause [2]. With the ageing population and rise in life expectancy worldwide, the stages of menopause and post-menopause will account for about one third of women’s lives [1]. Moreover, the number of post-menopausal women is also expected to increase. It is estimated that there will be 1.1 billion post-menopausal women in the world by 2025 [1]. Therefore, the health of this large population group is of high importance to public health.

Menopause is often accompanied by an increased risk for cardiovascular diseases (CVD) and sleep problems [3–5]. During the normal physiological process of menopause, the reduction in ovarian follicular function often leads to the depletion of estrogen, contributing to the development of a series of cardio-metabolic risks. These risks include central obesity, reduced glucose tolerance, increased blood pressure, abnormal levels of plasma lipids, and vascular inflammation [6], and as a consequence, substantially increasing the CVD risk and mortality in this group of women [7]. The hormonal changes that occur during menopause have also been reported to be correlated with various sleep problems [8]. The common complaints among post-menopausal women include difficulty in sleep onset, short sleep duration, and poor sleep quality [9]. It was reported that around 53.3% to 65.8% of post-menopausal women suffered from sleeplessness during their menopausal transition [10].

Numerous studies have demonstrated the associations between sleep disorders and increased CVD risk in general population [11]. An increased risk for CVD and coronary heart disease (CHD) incidents was found in individuals who had short sleep duration (≤ 6 h) and poor sleep quality [11]. Furthermore, in those who had sleep-disordered breathing (e.g., apnea and hypopnea), the relative risk for heart failure and stroke was 2.38 and 1.58, respectively [12]. However, limited studies examined the relationship of these health-related aspects particularly among post-menopausal women, and even fewer studies were conducted among Chinese post-menopausal women, the largest population group of this type in the world.

Previous studies among general populations usually assessed sleep quality by a single item, such as “How often is your sleep satisfactory?”, “How many hours of sleep do you usually get?” or “How often do you experience insomnia?” [11]. Moreover, limited evidence was obtained from a comprehensive and validated assessment of sleep quality. Thus, the present study aimed to investigate sleep quality using a comprehensive and validated tool and to examine the relationship between sleep quality and the CVD risk among Chinese post-menopausal women.

## Methods

### Study design and participants

This study was a secondary data analysis of a cross-sectional study conducted in Xi’an, Shaanxi Province, China. The original cohort consisted of 404 community-dwelling adults, whose sleep quality and its associated factors were examined [13]. The present study focused on the data of 154 post-menopausal women from the original cohort to evaluate their sleep quality and CVD risk. Post-menopause was defined as amenorrhea not attributable to other causes for at least 12 months [14]. The eligibility of the participants was assessed by their self-reported menstrual history.

### Measures

Data were collected by a structured questionnaire that included the Pittsburgh Sleep Quality Index (PSQI), items on cardiovascular parameters, and socio-demographic information. All questionnaires were administered by one researcher through face-to-face interviews.

#### Pittsburgh sleep quality index

Sleep quality was assessed by the Chinese-version of the PSQI (PSQI-C) [15]. The PSQI-C consists of 19 self-rated items related to sleep quality in the past month, of which seven component scores are summed to give a global score. The seven components include subjective sleep quality, sleep latency, sleep duration, habitual sleep efficiency, sleep disturbances, use of sleep medication, and daytime dysfunction. Each component score ranges from 0 to 3, and a score > 1 indicates problems in that aspect of sleep quality. The global score of sleep quality ranges from 0 to 21 with a higher score indicating poorer quality of sleep, whereas a cutoff score of ≤5 indicates good sleep quality [16]. The original PSQI had received extensive support to confirm its good psychometric properties and high correlation with actual sleep log data [17, 18]. Good reliability of the PSQI-C was also demonstrated among 793 Chinese adults, with a Cronbach’s alpha = 0.84 and a 2-week test-retest reliability of 0.81 [15].

#### Ten-year risk of CVD

##### Anthropometric measurements

Anthropometric measurements were administered in the same manner for all participants. Body weight and height were measured without shoes. Body mass index (BMI) was calculated as weight divided by the square of height (kg/m^2^). Systolic blood pressure (SBP) and diastolic blood pressure (DBP) were measured by a mercury sphygmomanometer after at least 15 min of rest.

##### Framingham 10-year risk score

The CVD risk in the participants was estimated by the office-based prediction model of the Framingham 10-year risk score (FRS) [19]. The predictors of the model include age, BMI, SBP, use of antihypertensive medication, medical history of diabetes, and current smoking status. This model revealed good performance in model discrimination and calibration, with a discrimination statistic of 0.785 [95% confidence interval (CI), 0.764–0.806] and a calibration Chi-square of 10.24 [19]. The sensitivity and specificity of this model for CVD events in follow-up studies were 0.58 and 0.83, respectively [19]. According to the American Heart Association guidelines for women’ health, a FRS ≥ 10% is regarded as being at high risk for CVD [7].

Socio-demographic variables included age, marital status, education level, employment status, medical history, and drinking and smoking status, which were collected based on self-report information. The participants’ physical exercise habit was evaluated by a single item: “How often do you do physical exercise?” A frequency of three times per week or above was regarded as a regular physical exercise habit [7].

### Statistical analysis

Descriptive statistics were used to describe the socio-demographic characteristics, cardiovascular parameters, and sleep quality of the participants. The normality of each continuous variable was tested through skewness and kurtosis statistics.

Multivariate regression and logistic regression were used to determine the association between sleep quality, socio-demographic variables, and CVD risk. To identify potential predicting variables encoded in the regression models, bivariate analyses between participants’ characteristics, sleep quality and FRS were conducted through independent t-test and Pearson correlation analyses as appropriate. As the FRS was calculated based on variables of age, gender, BMI, SBP, and smoking status [19], those variables were not examined in the bivariate analyses. A liberal significance level of 0.2 was applied to identify potential predictors, which would reduce the probability of excluding important variables from the model [20]. To reduce over-parameterization of the model, potential predictors with high co-variability (*r* > 0.7) and lowest correlation coefficient with the outcome variables were excluded from the model [20]. The two-sided level of significance was set at 0.05. Statistical analysis was performed by the IBM SPSS version 21.0 [21].

## Results

This study comprised of 154 Chinese post-menopausal women with a mean age of 63.65 (standard deviation, SD = 4.47) years. Table 1 summarizes the socio-demographic characteristics and cardiovascular parameters of the post-menopausal women. A majority of participants were married (81.8%) and had an education level of secondary or above (78.5%). For those who were still in employment (10.4%), most were employed as manual labourers, which required a high level of physically activity. The majority of the women (72.1%) had regular physical exercise. Hypertension was the most prevalent chronic illness (66, 43%), followed by diabetes (13%) and hypercholesterolemia (4%). Among those with hypertension, only 25 (37.9%) were taking antihypertensive medications. Only 1.3% of the participants were current smokers and 5.1% were drinkers. Their mean BMI was 24.41 (SD = 3.64) kg/m^2^, and 67.6% of the women were overweight or obese. The mean values for SBP and DBP were 126.20 (SD = 15.10) and 78.41 (SD = 7.92) mmHg, respectively. The participants had a mean 10-year risk of CVD of 12.54%, and half of them (51.3%) were at high risk for CVD (FRS ≥ 10%) [22, 23].

**Table 1:** Characteristics and cardiovascular parameters of the 154 post-menopausal women included in the analysis

Socio-demographic characteristics		Mean ± SD or *n* (%)
Age, years	(Range: 52–70)	63.65 ± 4.47
Marital status	Married	126 (81.8%)
	Widowed or divorced	28 (18.2%)
Educational level	Primary or less (≤ 6 years)	33 (21.4%)
	Secondary (7–12 years)	104 (67.5%)
	Tertiary or above (> 12 years)	17 (11.0%)
Employment status	Retired	138 (88.6%)
	With full-time/part-time job	16 (10.4%)
Medical history	Hypertension	66 42.9%)
	-Use of anti-hypertensive medications	25 (37.9%)
	Diabetes	20 (13.0%)
	Hypercholesterolemia	6 (3.8%)
With regular physical exercise		111 (72.1%)
Current smoker		2 (1.3%)
Current drinker		8 (5.1%)
Cardiovascular parameters		
Body mass index (BMI)^a^, kg/m^2^	(Range: 16.9–36.73)	24.41 ± 3.64
	Overweight (BMI: 23.0–24.9)	40 (26.0%)
	Obese (BMI ≥ 25.0)	64 (41.6%)
Systolic blood pressure, mmHg	(Range:90–180)	126.20 ± 15.10
Diastolic blood pressure, mmHg	(Range:59–100)	78.41 ± 7.92
Framingham 10-year risk score (FRS), %	(Range: 2.90–65.46)	12.54 ± 8.36
High risk FRS(≥10%)		79 (51.3%)

^a^The cutoff points for body mass index were selected based on the updated WHO expert consultation on ‘*Appropriate body mass index for Asian populations and its implications for policy and intervention strategies*’ in 2004

Findings from the PSQI showed that the sleep duration of the participants ranged from 1 to 9 (mean = 6.09, SD = 1.52) hours, with 25.0% of the women having less than 5 h sleep. Time spent in bed ranged from 5 to 13 (mean = 7.77, SD = 1.11) hours. The mean sleep efficiency, calculated by actual sleep duration / time spent in bed, was 78.35% (range: 7.69–100%). Table 2 presents the participants’ sleep quality, with regard to the seven component scores and the global score of PSQI. The global PSQI score was 8.58 ± 4.37. Six components, except for use of sleep medication, were scored >1. The component of sleep latency had the highest score (2.32), followed by sleep disturbance (1.51) and subjective sleep quality (1.26). Of the 154 participants, 136 (88.3%) reported “had to get up to use the bathroom” at least once per week as the most common cause of sleep disturbance, followed by “cannot get to sleep within 30 minutes” (*n* = 110, 71.4%), and “waking up in the middle of the night or early morning” (*n* = 97, 63.0%).

**Table 2:** Sleep quality of the Chinese post-menopausal women (*N* = 154)

Sleep quality		Mean ± SD or *n* (%)
Subjective sleep quality	(Range: 0–3)	1.26 ± 0.91
Sleep latency	(Range: 0–3)	2.32 ± 0.69
Sleep duration	(Range: 0–3)	1.15 ± 1.11
	0 (≥ 7 h)	58 (37.7%)
	1 (6–7 h)	41 (26.6%)
	2 (5–6 h)	29 (18.8%)
	3 (< 5 h)	26 (16.9%)
Habitual sleep efficiency	(Range: 0–3)	1.08 ± 1.19
Sleep disturbance	(Range: 0–3)	1.51 ± 0.61
Sleep medication	(Range: 0–3)	0.14 ± 0.54
Daytime dysfunction	(Range: 0–3)	1.10 ± 1.06
Global PSQI score	(Range: 0–18)	8.58 ± 4.37
Good sleep quality	Global PSQI ≤5	42 (27.3%)
Global PSQI score among groups of BP control^a^	Hypertension_controlled (*n* = 52)	9.77 ± 3.93
	Hypertension_uncontrolled (*n* = 14)	8.36 ± 3.56
	Non_hypertension (*n* = 88)	7.91 ± 4.62
Causes of sleep disturbances (≥ once per week)		
Cannot get to sleep within 30 min		110 (71.4%)
Waking up in the middle of the night or early morning		97 (63.0%)
Had to get up to use bathroom		136 (88.3%)
Cannot breathe comfortably		19 (12.3%)
Cough or snore loudly		70 (45.5%)
Feel too cold or hot		23 (14.9%)
Feel too cold or hot		48 (31.2%)
Having bad dreams		65 (42.2%)
Pain		61 (39.6%)
Other problems		57 (37.0%)

*PSQI* Pittsburgh sleep quality index

^a^ Criteria for blood pressure (BP) control follows the recommendations of Eighth Joint National Committee (JNC 8) that for those <60 years, the control target is systolic BP (SBP) < 140 mmHg and diastolic BP (DBP) < 90 mmHg; and for those ≥60 years, the control target is SBP < 150 mmHg and DBP < 90 mmHg. The one way analysis of variance revealed: F = 3.055, *p* = 0.050054

In the multivariate regression, the FRS was analyzed as the dependent variable. Bi-variate analyses were conducted to identify potential predicting variables of CVD risks. As presented in Table 3, five variables with a significance level < 0.2 in the bi-variate analyses with FRS, including subjective sleep quality (*r* = 0.138, *p* = 0.087), sleep duration (*r* = 0.106, *p* = 0.191), sleep disturbance (*r* = 0.264, *p* = 0.001), global PSQI score (*r* = 0.124, *p* = 0.126), and employment status (*t* = 6.414, *p* = 0.012). Given that global PSQI score was calculated based on all the seven component scores [15], to better understand the influences of specific sleep quality components on CVD risks, the variables of subjective sleep quality, sleep duration, and sleep disturbance, together with employment status were included as independent variables in the regression model. Moreover, physical exercise habit was also adjusted in the regression model, for its significant influence on CVD risk observed in the previous study [11]. The multivariate regression model (Table 4) identified three variables that influenced the CVD risk: employment status (*p* = 0.016), sleep duration (*p* = 0.04), and sleep disturbance (*p* < 0.001).

**Table 3:** Bivariate analyses between participants’ characteristics, sleep quality and Framingham 10-year risk score (*n* = 154)

Participants’ characteristics	Statistical values	*p*-value
Categorical variables^a^	*t*/F	
Marital status	*t* = 0.076	0.783
Educational level	F = 0.242	0.786
Employment status	*t* = 6.414	0.012
Physical exercise habit	*t* = 0.127	0.722
Current drinker	*t* = 1.561	0.722
Continuous variables^b^	*r*	
Subjective sleep quality	0.138	0.087
Sleep latency	0.103	0.203
Sleep duration	0.106	0.191
Habitual sleep efficiency	0.077	0.343
Sleep disturbance	0.264	0.001
Sleep medication	0.006	0.936
Daytime dysfunction	0.021	0.800
Global PSQI score	0.124	0.126

^a^Examined by independent t-test or one way analysis of variance

^b^Examined by Person correlation analyses. PSQI: Pittsburgh sleep quality index

**Table 4:** Multivariate regression model for the cardiovascular disease risk of the Chinese post-menopausal women^a^

Independent variables	β	95% CI	SE	*p*-value
Constant	–	(2.37, 3.23)	0.22	<0.001***
Sleep duration	- 0.18	(−0.32, −0.008)	0.08	0.04*
Sleep disturbance	0.33	(0.27, 0.85)	0.14	<0.001***
Employment status	- 0.19	(−1.15, −0.12)	0.26	0.016*
Physical exercise habit	0.02	(−0.31, 0.39)	0.18	0.83

Adjusted R^2^ = 0.110

^a^ The dependent variable was square rooted Framingham 10 year risk score

CI: confidence interval. SE: standard error. *p < 0.05; *** *p* < 0.001

Logistic regression analysis was also conducted to explore the relationship between sleep quality and CVD risk. The dependent variable is CVD risk, including a high-risk group (FRS ≥ 10%) and a low-risk group (FRS < 10%) [22, 23]. As indicated in the bivariate analysis with CVD risk, five variables (subjective sleep quality, sleep duration, sleep disturbance, global PSQI score, and employment status) revealed *p* values less than 0.2. As the global PSQI score has considered the scores on subjective sleep quality, sleep duration, and sleep disturbance. The independent variables in the logistic regress model included employment status and the sleep quality (including a good sleep quality group with PSQI ≤5 and a poor sleep quality group with PSQI >5). Given the significant influence on CVD risk [11], physical exercise habit was also adjusted in the logistic regression model. The results demonstrated that women with good sleep quality were less likely to be at high risk for CVD, with an odds ratio (OR) of 0.51 (95% CI: 0.26–0.97, *p* = 0.04).

## Discussion

The Chinese post-menopausal women had poor sleep quality and increased CVD risks. The current study also identified a significant relationship between sleep quality and CVD risk, which provided valuable clues for developing interventions to improve the sleep quality and to reduce the CVD risks in post-menopausal women.

The socio-demographic characteristics of the study sample were consistent with those in previous studies among Chinese post-menopausal women [24, 25]. The mean FRS in the study sample was 12.54%, indicating a high risk for CVD (> 10%) among the Chinese post-menopausal women. Previous studies have also reported an increased CVD risk among post-menopausal women, which may be related to the reduced protective effects of estrogen on cardiovascular health [8, 26].

The participants reported a poor sleep quality, with a mean global PSQI score of 8.58 (> 6). Their sleep quality was much poorer than that of other age groups of Chinese women in other studies [13, 24]. Particularly, poor sleep quality had been consistently reported in previous studies among post-menopausal women [10, 25]. These findings suggest that poor sleep quality is common among post-menopausal women in both western and Chinese populations.

On average, the participants spent 7.77 h in bed per night, but only had 6.09 h of sleep. The more than 1-h of awake in bed reflected a severe problem with sleep latency (mean score = 2.32) and may also have influenced their subjective perception of sleep quality [13]. Moreover, 71.4% of the participants reported “cannot get to sleep within 30 minutes” at least once per week. These findings are consistent with the high prevalence of insomnia reported in post-menopausal women in Asia [10, 24].

Most participants reported “have to get up to use bathroom,” “cannot get to sleep within 30 minutes,” and “waking up in the middle of the night or early morning.” as the most common causes of sleep disturbance. These sleep problems may be due to the aging changes of their urinary system, or to menopausal symptoms such as hot flashes and night sweats [13, 27]. Similar sleep problems were reported in the Wisconsin Sleep Cohort Study, in which post-menopausal women experienced more problems in initiating sleep (OR = 2.77) and repeated episodes of waking up (OR = 1.58) when compared with those of premenopausal women [27].

Sleep quality greatly affects the women’s quality of life and role functioning in work places and family [28]. The poor sleep quality observed among Chinese post-menopausal women indicates the urgent need for interventions to improve their sleep quality, and in particularly to address the long sleep latency and severe sleep disturbances.

The present study also identified that among the post-menopausal women, there was a significant relationship between the CVD risks and sleep quality, particularly in sleep duration and sleep disturbance. Women who slept longer (with a lower component score in sleep duration) were at higher risks for CVD. Previous studies reported a U-shaped relationship between sleep duration and health, suggesting that too little or too much sleep correlates with adverse health outcomes [29]. A meta-analysis of 15 prospective studies also reported that when compared to participants with normal sleep duration, those with longer sleep duration (> 8 h per night) had greater risks for total CVD incidents, CHD, and stroke [30]. The participants in the present study slept on average 6.09 h per night, and most (81.8%) had normal sleep duration with 5–8 h of sleep per night [30]. The present study added evidence that among the post-menopausal women with normal sleep duration, longer sleep duration was associated with higher CVD risks.

The participants who experienced more sleep disturbances had higher CVD risks. However, the relationship between sleep disturbance and CVD risk has received limited attention in previous studies. The sleep disturbances as measured by the PSQI included “cannot get to sleep within 30 minutes,” “waking up in the middle of the night or early morning,” “had to get up to use bathroom,” “cannot breathe comfortably,” “cough or snore loudly,” “feel too cold or hot,” “having bad dreams,” “pain,” and other problems. All these reported sleep disturbances may reflect the severity of some menopausal symptoms, such as urinary frequency, anxiety, and hot flashes. Moreover, the latter symptoms were significantly associated with increased CVD risks [5, 6, 31]. In addition, these sleep disturbances may have interrupted the physiological recovery function of sleep and therefore led to poorer cardiovascular health [11]. Future studies should attempt to develop CVD prevention strategies by addressing the sleep disturbances experienced by post-menopausal women.

Interestingly, women who were still working were at lower risk for CVD (*β* = −0.19, *p* = 0.016**)**. Some women might stop working due to the comorbidity of hypertension, diabetes, or poor sleep quality. These health problems would contribute to an increased CVD risk. On the other hand, the majority of the women in the working group were manual labourers, who are likely to have higher levels of physical activity compared with those in the non-working group. Moreover, the working women were significantly younger than the non-working women (57.8 vs. 64.3 years, *p* < 0.001). Because age is a significant predictor in the algorithm of the FRS [19], the correlation between working status and CVD risk should be interpreted with caution. Future studies should pay attention to promote the cardiovascular health among post-menopausal women who are not working, such as improving their physical activity levels.

The CVD risk was found to be reduced by half in women who had better sleep quality than those who had poor sleep quality (OR = 0.51, *p* = 0.04). This finding was consistent with the results of previous studies. Good sleep quality has been recognized as a beneficial factor for cardiovascular health, and may prevent the development of CVD [9]. One study in the Netherlands reported that short-duration sleepers with poor sleep quality had a 63% increased risk for CVD, and a 79% higher risk for CHD when compared to normal-duration sleepers who had good sleep quality [11]. This evidence further strengthens the need to include sleep quality as an important indicator when assessing CVD risk among Chinese post-menopausal women.

Considering the critical contribution of SBP and use of anti-hypertensive medication on CVD risks and the close relationship between sleep quality and CVD risks in the current study participants, further analysis was conducted to explore the relationship between sleep quality and BP control. The Eighth Joint National Committee recommended that for those <60 years, the control target is SBP < 140 mmHg and DBP < 90 mmHg; and for those ≥60 years, the control target is SBP < 150 mmHg and DBP < 90 mmHg [32]. Accordingly, the participants were divided into three groups: those with hypertension and controlled BP; those with hypertension but uncontrolled BP; and a group without hypertension. However, the one way analysis of variance did not revealed statistically significant difference in sleep quality among the groups (F = 3.055, *p* = 0.050054).

To the best of our knowledge, this is the first study to examine the relationship between sleep quality and CVD risk among Chinese post-menopausal women. Nonetheless, this study had some limitations. First, this was a cross-sectional study with an internal limitation in confirming the cause-and-effect relationships. Future study should use a prospective study design with long-term follow-up to further explore the relationship between sleep quality and CVD risk. Secondly, sleep quality was measured by the self-reported PSQI. Previous studies had extensively used such a self-report system, even through a single item to assess sleep duration or sleep quality. Anyway, the PSQI is a comprehensive assessment of sleep quality and has revealed good performance in psychometric properties [27, 30]. To eliminate the possibility of self-reporting bias, future studies should employ a more objective approach to measure sleep quality, such as polysomnography and actigraphy. Moreover, the medical history of hypertension, diabetes, and hypercholesterolemia was also collected based on participants’ self-report information. To get more accurate information, future studies could conduct blood tests or retrieve data from participants’ medical records.

## Conclusions

In conclusion, this study identified that Chinese post-menopausal women had poor sleep quality and increased CVD risk. Special attention should be paid to improve the sleep quality and cardiovascular health in these women. As indicted by the significant relationship between sleep quality and CVD risk, poor sleep quality might increase the CVD risk in post-menopausal women. To improve their cardiovascular health, future interventions should target towards strategies for improving sleep quality and for addressing sleep disturbances.

## Abbreviations

BMI: Body mass index
CHD: Coronary heart disease
CI: Confidence interval
CVD: Cardiovascular disease
DBP: Diastolic blood pressure
FRS: Framingham 10-year risk score
OR: Odds ratio
PSQI: Pittsburgh sleep quality index
SBP: Systolic blood pressure
SD: Standard deviation
